# The regulation of self-tolerance and the role of inflammasome molecules

**DOI:** 10.3389/fimmu.2023.1154552

**Published:** 2023-04-04

**Authors:** Qi Ke, Ashley Nicole Greenawalt, Veera Manukonda, Xingqi Ji, Roland Michael Tisch

**Affiliations:** ^1^ Department of Microbiology and Immunology, University of North Carolina at Chapel Hill, Chapel Hill, NC, United States; ^2^ Lineberger Comprehensive Cancer Center, University of North Carolina at Chapel Hill, Chapel Hill, NC, United States

**Keywords:** autoimmunity, self-tolerance, inflammasomes, immunoregulation, inflammation

## Abstract

Inflammasome molecules make up a family of receptors that typically function to initiate a proinflammatory response upon infection by microbial pathogens. Dysregulation of inflammasome activity has been linked to unwanted chronic inflammation, which has also been implicated in certain autoimmune diseases such as multiple sclerosis, rheumatoid arthritis, type 1 diabetes, systemic lupus erythematosus, and related animal models. Classical inflammasome activation-dependent events have intrinsic and extrinsic effects on both innate and adaptive immune effectors, as well as resident cells in the target tissue, which all can contribute to an autoimmune response. Recently, inflammasome molecules have also been found to regulate the differentiation and function of immune effector cells independent of classical inflammasome-activated inflammation. These alternative functions for inflammasome molecules shape the nature of the adaptive immune response, that in turn can either promote or suppress the progression of autoimmunity. In this review we will summarize the roles of inflammasome molecules in regulating self-tolerance and the development of autoimmunity.

## Introduction

A functioning immune system is characterized by the capacity to distinguish between self-antigens versus microbial pathogens and foreign molecules. Several mechanisms are in place regulating both innate and adaptive immunity to establish persistent self-tolerance. These mechanisms maintain self-tolerance by limiting the activation and maturation of innate effectors such as monocytes, macrophages and dendritic cells (DC), while regulating self-specific T and B cells *via* intrinsic and extrinsic events. Immunoregulation is a dominant mechanism by which self-tolerance is established and maintained. Multiple subsets of self-specific T cells, including forkhead box P3 (FoxP3)-expressing regulatory CD4^+^ T cells (Foxp3^+^Treg), as well as regulatory B cells, mediate immunoregulation *via* 1) secretion of anti-inflammatory cytokines (e.g. TGF β1, IL-10) and modulatory factors, and 2) cognate interactions with T and B cells and/or DC and macrophages serving as antigen-presenting cells (APC) by engagement of various ligand-receptor molecules. Subsets of DC and macrophages also contribute to immunoregulation through secretion of cytokines and factors. Breakdown of self-tolerance leads to autoimmunity, typically characterized by chronic inflammation driven by autoreactive T and B cells, autoantibodies, and/or activated macrophages, DC and other innate effectors (1). Autoimmune diseases are characterized as: 1) organ-specific autoimmunity, such as multiple sclerosis (MS), rheumatoid arthritis (RA) and type 1 diabetes (T1D), or 2) systemic autoimmune diseases, such as systemic lupus erythematosus (SLE) (2). Events leading to the failure of self-tolerance are complex and influenced in a polygenic manner, while involving a host of ill-defined environmental factors including microbial infections, toxins, ultraviolet (UV) irradiation, diet, and dysbiosis of the gut microbiota.

The immune system has also evolved to detect and rapidly respond to invading pathogens *via* innate cell-driven events. This early inflammation leads to subsequent expansion and differentiation of effector T and B cells, typically resulting in clearance of the pathogen, and establishment of long-lasting immune protection. Recognition of an invading microbial pathogen is mediated by surface and cytoplasmic pattern recognition receptors (PRRs) which recognize: 1) conserved pathogen-associated molecular patterns (PAMPs), and 2) endogenous-derived danger-associated molecular patterns (DAMPs) induced by tissue damage and cellular activities mediated by microbial virulence factors (3).

Inflammasomes are oligomeric complexes that play an important role in initiating inflammation in response to PAMPs and DAMPs (4). Appropriately regulated activation of inflammasomes protects against microbial infection. However, aberrant inflammasome activity has been associated with severe inflammation-driven pathologies (5–7), as well as autoinflammatory and autoimmune diseases (8). Notably, inflammasome receptor molecules regulate the properties of different immune cell effectors as well as non-immune cell types that is independent of classical activation and inflammation-inducing events (9). This alternative function of inflammasome molecules has also been directly linked to autoimmunity and sterile inflammation. In this review, we will discuss how inflammasomes contribute to autoimmunity: 1) by inflammation driven by classical inflammasome activation, and 2) *via* alternative functions inflammasome molecules display.

## Inflammasome-mediated inflammation- an overview

**Figure 1:**
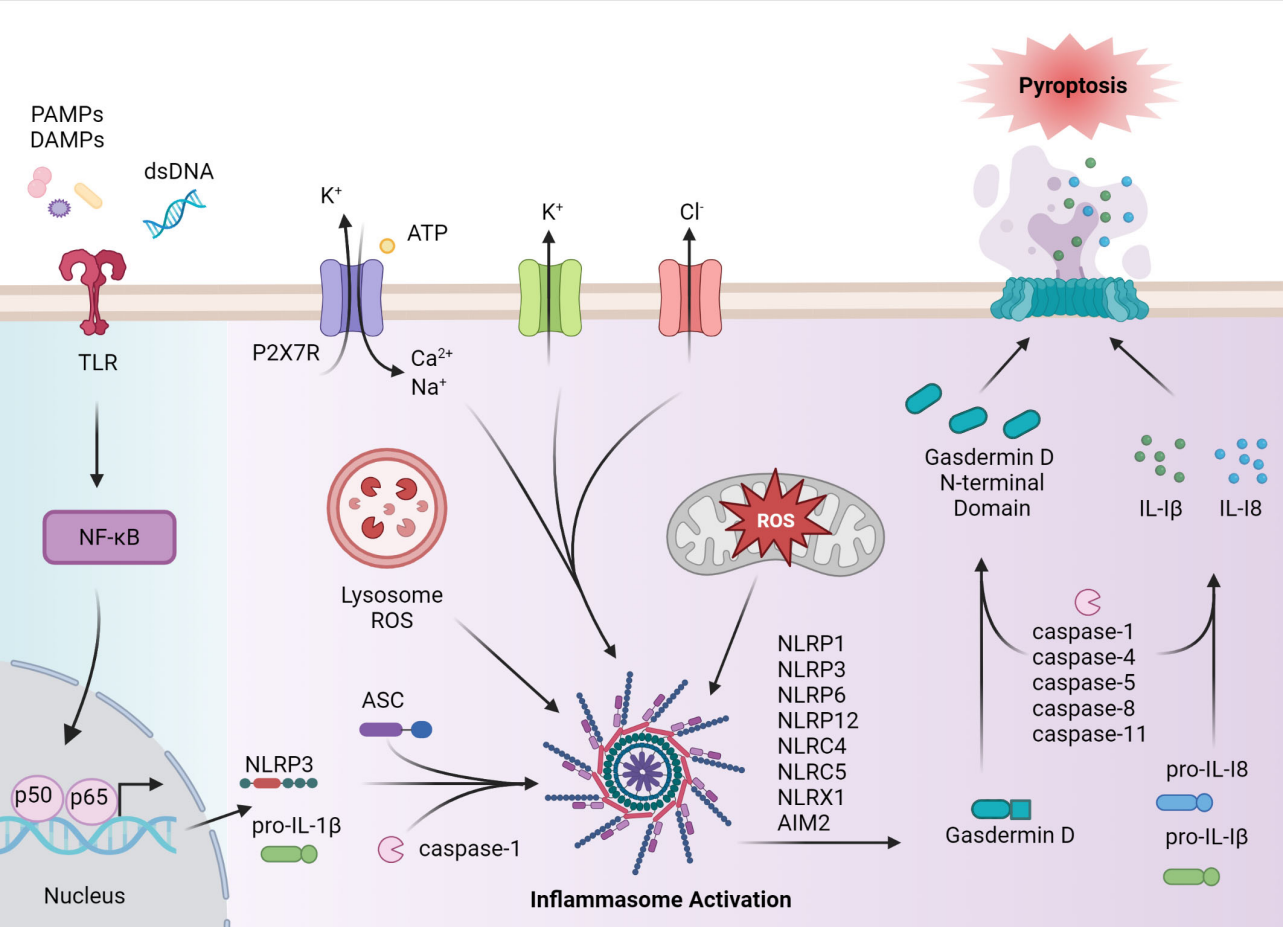
Inflammasome assembly and activation. Canonical activation of the inflammasome pathway begins with a primary signal, such as PAMPs, endogenous-derived DAMPs, or dsDNA, that are recognized by pattern recognition receptors (PRRs), such as toll-like receptors (TLRs). PRR activation induces NF-κB and subsequent expression of NLRP, pro-IL-1β and pro-IL-18, and post-translational events. Formation of the inflammasome complex occurs when the sensor protein, such as NLRP3, binds to ASC, driving caspase activation and inflammasome assembly. Caspase enzymes cleave pro-IL-1β and pro-IL-18 as well as the C terminus from gasdermin D, allowing the gasdermin D N-terminal domain to form pores necessary for pyroptosis. IL-1β and IL-18, as well as cellular contents are released to establish a proinflammatory response. In autoimmune disease, inflammasome activation can occur *via* activation in a noncanonical matter including agonist-induced ion flux and lysosomal and mitochondrial reactive oxygen species (ROS). The figure was prepared using Biorender software licensed to the UNC Lineberger Comprehensive Cancer Center.

Inflammasome-driven inflammation in the context of innate immunity generally entails the production of proinflammatory cytokines such as IL-1β and IL-18, as well as induction of programmed cell death. The typical inflammasome complex consists of three components; namely 1) a sensor molecule such as a nucleotide oligomerization domain-like receptor (NLR), Absent in melanoma 2-like receptors (ALR) or pyrin, 2) the adaptor molecule apoptosis-associated speck-like protein (ASC) that contains a caspase activation and recruitment domain (CARD), and 3) pro-caspase-1 (**Figure 1**) (4). The assembled inflammasome provides a platform for cleavage of pro-caspase-1 (4). Once activated *via* an autolytic processing event, caspase-1 mediates maturation of pro-IL-1β and pro-IL-18 precursors, as well as initiating pyroptosis (4).

Pyroptosis, a lytic form of programmed cell death, is induced through caspase-1-mediated cleavage of gasdermin D (GSDMD), which removes the autoinhibitory C-terminus portion of the protein (10). Cleaved GSDMD also forms pores in the cell membrane, which facilitate the secretion of mature IL-1β and IL-18 (11). Cleavage of GSDMD and induction of pyroptosis is also achieved by a nonconical pathway in which murine caspase-11 or human caspase-4/5 are activated by cytosolic lipopolysaccharide (LPS), a gram-negative bacteria endotoxin (11, 12). In addition to pyroptosis, certain inflammasome molecules such as NLR family pyrin domain containing 3 (NLRP3) and absent in melanoma 2 (AIM2), have been associated with PANoptosis-driven cell death in response to microbial infection and changes in cellular homeostasis (13). PANoptosis is regulated by the PANoptosome, which is a multimeric complex consisting in part of effector molecules involved in pyroptotic (caspase 1), apoptotic (caspase 8), and necroptotic (receptor-interacting protein kinase 1 (RIPK1), receptor-interacting protein kinase 3 (RIPK3)) cell death pathways (14). The composition of the PANoptosome varies with the nature of the stimulatory response, and complexes consisting of the ASC adaptor and NLRP3 or AIM2 sensor molecules have been identified (15).

Inflammasome activation is achieved in response to a broad range of stimuli derived from microbial infection, tissue damage, and/or dysregulation of metabolic events (**Figure 1**). The process of inflammasome activation typically entails two sets of signaling events that prime (signal 1), and activate (signal 2) the inflammasome (11). This multiple-step pathway ensures robust regulation of inflammasome activity. Signal one, induced by PRR (e.g. toll-like receptors (TLR)) primes inflammasome assembly *via* activation of NF-κB, upregulation of pro-IL-1β and pro-IL-18 expression, and induction of post-translational events that favor the formation of an inflammasome complex (11, 12). Signal two is specific for a given sensor molecule and induces inflammasome activation (12). Binding of an agonist to the leucine-rich repeat containing receptor (LRR) portion of the sensor protein leads to oligomerization *via* homotypic pyrin (PYD) interactions with the ASC adaptor molecule. ASC is important for linking the sensory protein with caspase-1 *via* CARD interactions (11, 12). Events driving caspase-1 activation, IL-1β and IL-18 maturation, and induction of pyroptosis and/or PANoptosis then follow (11, 12). To date, the role of inflammasomes in autoimmunity have largely focused on NLRP3 and AIM2, but other inflammasome molecules such as NLRP1, and NLR family CARD domain-containing protein 4 (NLRC4) have also been implicated in autoimmunity (16, 17). The respective inflammasomes are defined by the sensor protein.

NLRP3 has been the most extensively studied inflammasome, in general and in autoimmunity (18). NLRP3 agonists are structurally and chemically diverse: such agonists include 1) PAMPs expressed by bacteria, virus, and fungi, and 2) DAMPs including cholesterol, extracellular ATP, microbial pore-forming toxins, and particulate matter such as uric acid crystals (19). Consequently, it is believed that these agonists are indirectly sensed by NLRP3. Here, agonist-induced K^+^ and Cl^-^ effluxes, Ca^2+^ fluxes, lysosomal damage, and mitochondrial damage and/or dysfunction coupled with the release of reactive oxygen species (ROS) are directly sensed by NLRP3 (20). For instance, noncanonical-induced activation of GSDMD results in K^+^ efflux, which activates NLRP3 and leads to caspase-1-mediated IL-1β and IL-18 production *via* the classical pathway (21–23). Gain of function variants in the *NLRP3* gene resulting in aberrant NLRP3 inflammasome activation cause a family of diseases referred to cryopyrin-associated periodic syndromes (CAPS), which are marked by reoccurring systemic inflammation (20). NLRP3 activation has also been linked to diseases of the central nervous system (CNS) such as Alzheimer’s Disease (AD) (24, 25). In AD, the accumulation and subsequent uptake of amyloid-β by microglia residing in the brain results in lysosomal destabilization and NLRP3 activation (24). Production of IL-1β also has neurotoxic effects on microglia and astrocytes (25).

The process of NLRP1 activation is distinct from other inflammasomes (26). Here, motif-dependent ubiquitination followed by degradation of the N-terminal subunit by proteasome are required for activation of NLRP1 (27, 28). Various bacterial toxins and viral proteases have been reported to activate NLRP1 in mice and humans (29–33). However, since mice encode several NLRP1 orthologues with sequences that differ from the single human encoded *NLRP1* gene, specific PAMPs and DAMPs triggering NLRP1 activation are variable and not fully defined among the species (34–37). The NLRC4 inflammasome is also distinct compared to other inflammasomes, in which the sensor protein functions as an agonist receptor. Instead, the NLRC4 protein associates with NLR family apoptosis inhibitory proteins (NAIPs) that act as cytosolic innate immune receptors, and which bind bacterial flagellin and type III secretion system components (T3SS) (38, 39). Gain-of-function variants in *NLRC4* lead to periodic fever syndromes marked by increased systemic IL-18 (40).

AIM2 is responsive to cytosolic double-stranded DNA (dsDNA) from bacteria and DNA viruses. Notably, AIM2 binds both endogenous and microbe-derived dsDNA independent of nucleic acid sequence (41). Expression of AIM2 is upregulated by type I interferon (IFN), and the AIM2 inflammasome is key in host defense against bacterial and viral pathogens such as *Francisella tularensis* and *Listeria monocytogenes*, and vaccinia virus and cytomegalovirus, respectively (42). In addition, the AIM2 inflammasome promotes caspase-1-driven death of intestinal epithelial cells and hematopoietic bone marrow cells upon recognition of dsDNA breaks due to ionizing radiation or chemotherapeutic drugs (43).

## The roles of IL-1β and IL-18 in inflammation

Inflammasome generated IL-1β and IL-18 enhances both innate and adaptive immunity against microbial pathogens. However, dysregulated production of these two cytokines by inflammasomes is also linked to chronic autoimmune diseases.

IL-1β is produced largely by monocytes, macrophages, and DC (44). Local release of IL-1β amplifies inflammation by inducing increased expression of 1) adhesion molecules and chemokines for recruitment of immune effectors, as well as 2) proinflammatory mediators such as cyclooxygenase type 2 (COX-2) and prostaglandin-E2 (PGE2) (44–46). IL-1β production can also lead to systemic inflammation *via* induction of the acute phase response, vasodilatation, angiogenesis, and leukocyte activation (44, 45).

T cell responses are also regulated both indirectly and directly by IL-1β. For instance, IL-1β enhances the stimulatory capacity of DC by driving maturation and upregulation of co-stimulatory molecules needed for efficient T cell activation and expansion (47). Increased IL-12 secretion by IL-1β stimulated DC favors differentiation of antigen-stimulated T cells towards a type 1 phenotype, marked by IFNγ production by CD4^+^ Th1 and CD8^+^ Tc1 cells (48). On the other hand, IL-1β has direct effects on CD4^+^ and CD8^+^ T cells, influencing expansion and subset differentiation depending on the extracellular *milieu* (49). In mice, IL-1β synergizes with IL-6, IL-21 and IL-23 to induce the differentiation of CD4^+^ T cells into IL-17-secreting Th17 cells (49). In humans, IL-1β has a more potent role in driving Th17 differentiation. Both Th1 and Th17 cells play key roles in several autoimmune diseases. Furthermore, IL-1β can suppress the function and/or reduce the stability of Foxp3^+^Treg (50, 51). Dysregulation of the Foxp3^+^Treg pool leading to skewed differentiation and pathogenic function of autoreactive effector T cells (Teff) is associated with a number of autoimmune diseases (52–56). CD8^+^ T cell expansion and differentiation are also regulated by IL-1β (57).

IL-1β has regulatory effects on the B cell compartment by enhancing B cell proliferation and antibody production (45). In addition, IL-1β increases proliferation and secretion of IL-4 and IL-21 by CD4^+^ T follicular helper cells (Tfh) (58). Tfh cells play a critical role in regulating antibody production by B cells and have also been implicated in the production of autoantibodies during autoimmunity (59).

IL-18 is expressed by a variety of cells such as Kupffer cells, macrophages, DC, and non-hematopoietic cells that include intestinal epithelial cells, keratinocytes and endothelial cells (60). Locally, IL-18 stimulates myeloid and endothelial cells to upregulate nitric oxide (NO) synthesis, and expression of cell adhesion molecules and chemokines to recruit and activate additional immune effectors at the site (60). In addition, IL-18 has potent regulatory effects on T cells and natural killer (NK) cells (60). IL-18 along with IL-12 drives the differentiation of Th1 cells and induces IFNγ production by CD8^+^ T cells and NK cells (60, 61). Furthermore, IL-18 stimulation upregulates 1) perforin- and Fas ligand (FasL)-dependent cytotoxicity in CD8^+^ T cells and NK cells, and 2) IL-17 secretion by γδ T cells (62). Not only is IL-18 linked to autoimmune diseases such as T1D and SLE, IL-18 has also been shown to play a key role in the maintenance of the intestinal epithelial barrier and regulation gut microbiota composition (63, 64). Dysbiosis of gut microbiota has been suggested as a risk factor for the development of autoimmunity (65, 66).

## Classical inflammasome activation-dependent events in autoimmunity

In view of highly potent proinflammatory effects, it is not surprising that classical inflammasome activation is linked to a host of autoimmune diseases. Inflammasome activation is detected in innate and adaptive immune effectors thereby having indirect and direct effects that shape and maintain the proinflammatory response either locally and/or systemically in autoimmunity. In addition, inflammasome activation in non-immune cell types that makeup a given organ can initiate and/or exacerbate an autoimmune response. Finally, evidence indicates that inflammasome activation can have a protective role and contribute to maintenance of self-tolerance. In the following, we will describe the different roles classical inflammasome activation has in common tissue-specific and systemic autoimmune diseases (**Table 1**).

**Table 1:** Intrinsic-effects of classical inflammasome-mediated inflammation in autoimmunity.

Autoimmune Disease	Associated environmental trigger events	Genetic variants involved in inflammasome pathways	Inflammasome intrinsic effects on innate immune cells	Inflammasome intrinsic effects on adaptive immune cells	Inflammasome intrinsic effects on non-immune/tissue resident cells
**MS**	Epstein-Barr virus (EBV), human herpes virus 6 (HHV-6), human endogenous retrovirus (HERV), cytomegalovirus (CMV), varicella zoster virus (VZV) (67, 68) *Helicobacter pylori, Chlamydia pneumoniae, Staphylococcus aureus* (69) Mouse hepatitis virus (MHV) (70), Semliki Forest virus (SFV) (71)	*NLRP1*: p.G587S (72), Gly587Ser (73), p.Ile601Phe, p.Ser1387Ile (74) *NLRP3*: Q705K (75), p.Leu832Ile (74) *NLRC4*: p.Arg310Ter, p.Glu600Ter (74) *NLRP9*: rs10423927 (76)	Microglia: ↑NLRP3, NLRC4 (77), ↑NLRP9 (76), ↓NLRX1 (78, 79), ↓NLRP12 (80, 81) PBMC: ↑Caspase-1 (82) DC/macrophage: ↑NLRP3 (83), ↓NLRC3 (84) Peripheral myeloid cells: ↑ GSDMD (85)	T cells: ↑ASC (86), ↓NLRP12 (87, 88)	Oligodendrocytes: ↑Caspase-1 (89) Astrocytes: ↑NLRP3, ↑NLRC4 (77), ↓NLRX1 (90)
**RA**	*Porphyromonas gingivalis*, *Prevotella nigrescens, Tannerella forsythensis, Prevotella intermedia* (91, 92) *Aggregatibacter actinomycetemcomitans, Treponema denticola* (93) Decreases in α-diversity (94, 95)	*NLRP3*: rs10754558 (96), rs4612666 (97, 98)	Monocytes/macrophages/DC: ↑NLRP3, ↑ASC, ↑caspase-1 (99–104), ↑NLRC4 (105), ↑AIM2 (106, 107) Neutrophil: ↓NLRP3 (108)	T cells: ↑NLRP3 (109), ↓NLRP12 (110)	FLS: ↑AIM2 (111), ↓NLRP6 (112), ↑NLRP3 (113) (114), ↑NLRC5 (115),
**T1D**	Enteroviruses (116), *Mycobacterium avium* subspecies *paratuberculosis* (117)	*NALP1*: rs12150220 (118), rs11651270, rs2670660 (119) *NLRP3*: rs10754558 (120), rs3806265, rs4612666 (121) *NLRC4*: rs212704, rs385076 (122)	APC: ↑NLRP3 (123)		β cells: ↑NLRP3 (123) Intestinal tissues: ↓AIM2 (124), ↑NLRP3 (125, 126)
**SLE**	EBV, parvovirus B19 (B19V), HERVs (127) Gut virome (128) Dysbiosis in gut microbiota (129) Dysbiosis of oral microbiota (130)	*NLRP1*: rs12150220 (131), rs2670660 (131) *NLRP3*: rs4612666, rs10754558, rs6672995, rs3806268, rs35829419, rs4352135 (132)	Macrophages/PBMC/monocytes: ↑NLRP3 (133–137), ↑AIM2 (135, 138, 139)	Tfh: ↓P2X7R and GSDMD-induced pyroptosis (140)	Glomerular podocytes: ↑ NLRP3 (141, 142)

Multiple sclerosis (MS); rheumatoid arthritis (RA); type 1 diabetes (T1D); systemic lupus erythematosus (SLE); experimental autoimmune encephalomyelitis (EAE); antigen presenting cells (APC); dendritic cells (DC); fibroblast-like synoviocytes (FLS); germinal center (GC); human peripheral blood mononuclear cells (PBMC); interferon (IFN).

“↑” indicates increased activity of a given molecule. “↓” indicates reduced activity of a given molecule.

### Multiple sclerosis and inflammasome-mediated neuroinflammation

MS is a demyelinating autoimmune disease marked by chronic inflammation of the CNS, leading to variable neurological symptoms and heterogenous clinical outcomes (143, 144). MS susceptibility and disease progression are influenced by both genetic and environmental factors (145). Although ill-defined, the autoimmune response in MS is believed to be initiated in the periphery, involving stimulation of CD4^+^ and CD8^+^ T cells specific for myelin proteins (146, 147). Differentiation of the encephalitogenic CD4^+^ T cell pool is skewed towards Th1 and Th17 subsets. This pool coupled with CD8^+^ T cells and B cells migrate across the CNS microvascular endothelium and into the brain and spinal cord (148, 149). The CNS infiltrate includes peripheral monocytes/macrophages and DC that further amplify the autoimmune response. Upon activation, microglia, which are tissue-resident macrophages as well as resident astrocytes also contribute to inflammation (144, 150) by production of: 1) proinflammatory cytokines such as IL-1β, which has neurotoxic and immunomodulatory effects in the CNS, as well as 2) chemokines that promote recruitment of immune effector cells (151, 152).

**Figure 2:**
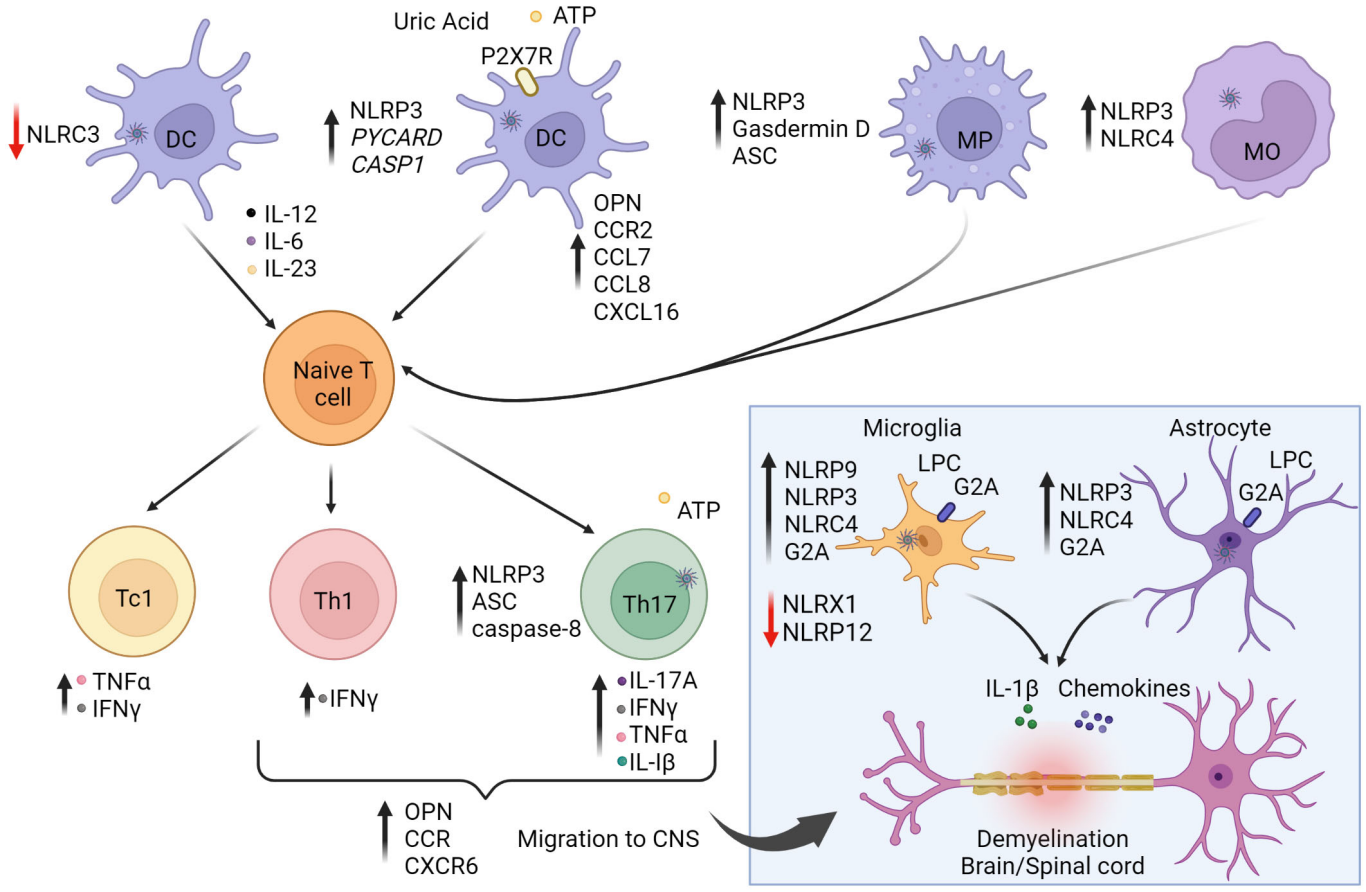
The role of inflammasomes in multiple sclerosis (MS) and experimental autoimmune encephalitis (EAE).** **The autoimmune response for MS is believed to begin in the periphery. Activation of NLRP3 and NLRC4 inflammasome pathways in antigen-presenting cells (APC) enhance stimulation and differentiation of pathogenic CD4^+^ Th1/Th17 and CD8^+^ Tc1 subsets. On the other hand, NLRC3 activation in dendritic cells (DC) is protective against disease by inhibiting DC maturation. Secretion of IL-1β and IL-18 increase T cell expression of osteopontin (OPN), CCR2 (binding CCL7/8), and CXCR6 (binding CXCL16) to promote infiltration to the central nervous system (CNS). Upon activation and differentiation, CD4^+^ and CD8^+^ T cells, and B cells migrate to the CNS. In the CNS, peripheral DC, macrophages (MP), and monocytes (MO) further amplify inflammation. CNS resident cells such as microglia and astrocytes also promote inflammation. Lysophosphatidylcholine (LPC) activates NLRP3 and NLRC4, causing secretion of IL-1β and chemokines, leading to further inflammation and demyelination. NLRP9 expression is increased in microglia. NLRX1 and NLRP12 serve to down-regulate neuroinflammation and provide protection against disease as indicated by the red arrows. Reduction of NLRX1 and NLRP12 can lead to exacerbated disease states. Purinergic receptor (P2X7R). The figure was prepared using Biorender software licensed to the UNC Lineberger Comprehensive Cancer Center.

Studies of MS patients and rodent experimental autoimmune encephalomyelitis (EAE), a model of MS, demonstrate that inflammasomes such as NLRP3, are associated with various aspects of the autoimmune process (153–155) (**Figure 2**). mRNA expression of *NLRP3* and *IL1B* are detected in MS lesions as well as increased levels of IL-1β and IL-18 in blood and cerebrospinal fluid (CSF) (150, 156). Furthermore, the P2X7 purinergic receptor (P2X7R), a ligand-gated ion channel regulated by extracellular ATP that activates the NLRP3 inflammasome (157), is elevated in the spinal cord of MS patients. Indeed, increased extracellular levels of ATP and uric acid are found in the CSF and serum of MS patients (158, 159). ATP is normally abundant in the extracellular space of the CNS, where it functions as an excitatory neurotransmitter. Interestingly, various drugs used to clinically treat MS such as recombinant IFNβ, glatiramer acetate and natalizumab suppress *NLRP3* mRNA expression, and decrease IL-1β in the blood and CSF of MS patients (160–162). In the brain lesions of MS patients, NLRP9 protein is also up-regulated in microglia but not astrocytes, suggesting a role for NLRP9 in modulating the encephalitogenic response (76).

The functional role of inflammasomes and inflammasome-related molecules has been investigated using EAE and other demyelinating rodent models. Earlier studies have shown that the progression and severity of EAE are reduced in mice deficient in NLRP3 (NLRP3^-/-^), ASC (ASC^-/-^) and to a lesser extent caspase-1 (Caspase1^-/-^) (83, 163). Attenuated EAE in NLRP3^-/-^ and ASC^-/-^ mice coincides with decreased infiltrates of Th1 and Th17 cells, macrophages and DC in the brain and spinal cord (83). This reduction in CNS infiltration is attributed to decreased production of IL-1β and IL-18 by APC (83). The latter are needed to adequately activate and upregulate T cell expression of osteopontin (OPN), and chemokine receptors CCR2, and CXCR6 for efficient migration into the CNS (83). In addition, lack of NLRP3 and ASC expression also limits DC and macrophages to upregulate matching receptor/ligands for OPN (α4β1 integrin), CCR2 (CCL7/CCL8), and CXCR6 (CXCL16) (83), resulting in aberrant APC migration into the CNS. These findings support a role for APC-expressed NLRP3 in mediating chemotactic recruitment of immune effectors to the CNS.

Peripheral APC also regulate the progression of EAE *via* inflammasome-mediated pyroptosis. EAE is attenuated in mice lacking GSDMD expression by peripheral myeloid cells (85). On the other hand, selective deletion of GSDMD in microglia has no effect on EAE, indicating that pyroptosis of CNS-resident APC may have only a limited role. The T cell stimulatory capacity of GSDMD^-/-^ APC is reduced, which is marked by diminished numbers and effector function of Th1 and Th17 cells in the CNS. Notably, selectively blocking GSDMD-mediated pyroptosis with the inhibitor disulfiram, also attenuates EAE, demonstrating a direct role for pyroptosis (85). It is believed that pyroptosis of APC heightens local inflammation to promote efficient T cell activation, and subset differentiation needed to generate a robust encephalitogenic T cell pool.

In addition to APC, inflammasome activity intrinsic to T cells impacts EAE progression (**Figure 2**). Selective ASC-deficiency in T cells attenuates EAE marked by reduced infiltration of CD4^+^ T cells, B cells, and neutrophils (86). ASC^-/-^ T cells are readily activated and undergo normal *in vitro* and *in vivo* differentiation into Th1, Th2, Th17 and Foxp3^+^Treg subsets. However, ASC-deficiency affects the properties of Th17 but not Th1 cells. ASC^-/-^ Th17 exhibit reduced survival and pathogenicity reflected by decreased secretion of IL-17A, IFNγ, TNFα, as well as IL-1β. Here, IL-1β plays a key role in an autocrine manner, by enhancing the survival and effector function of Th17 cells residing in the CNS. Interestingly, cleavage of pro-IL-1β in Th17 cells is mediated *via* a noncanonical pathway involving caspase 8 activation. In this scenario, increased extracellular ATP levels due to release by stressed and dying cells drives activation of the NLRP3-ASC-caspase-8 complex, establishing a feed-forward loop promoting Th17 cell-mediated pathogenicity.

In addition to NLRP3, the activity of other inflammasome molecules in non-immune CNS resident cell-types have been found to promote neuroinflammation. Both NLRP3 and NLRC4 regulate the activity of microglia and astrocytes in a cuprizone model of inflammation-induced demyelination (77). Both cell types are known mediators of neuroinflammation through secretion of proinflammatory cytokines and chemokines. Cuprizone-induced pathology is prevented in NLRP3- and NLRC4-deficient mice characterized by microglia and astrocytes lacking IL-1β production, and exhibiting reduced expression of G2A, the receptor for lysophosphatidylcholine (LPC) (**Figure 2**). LPC, known for proinflammatory properties, is rapidly metabolized under homeostasis but accumulates under pathological conditions in the CNS (77). Following cuprizone treatment, LPC levels are increased, and LPC functioning as a DAMP, activates NLRP3 and NLRC4 expressed by microglia and astrocytes (77). In MS patients, expression of G2A and NLRC4 are increased, suggesting a role in the MS autoimmune response (77).

Interestingly, inflammasomes have also been shown to play a protective role in EAE. For instance, deficiency of NLRC3 exacerbates EAE (84). Lack of NLRC3 results in DC producing increased proinflammatory cytokines such as IL-12, IL-6, and IL-23, that in turn enhance differentiation of encephalitogenic Th1 and Th17 cells (84). NLRC3 negatively regulates DC maturation by inhibiting activation of the p38 signaling pathway (84). The ligand(s) regulating NLRC3 activity in DC is currently undefined (84). Also serving a protective function is NLR family member X1 (NLRX1), a more recently characterized NLR that is ubiquitously expressed and located in the mitochondria (78, 90). NLRX1 inhibits proinflammatory pathways, including type I IFN and TLR-mediated NF-κB signaling events, and may play a role in regulating mitochondria oxidative damage (78). Mice deficient of NLRX1 have increased T cell infiltration of the CNS, and consequently develop more severe EAE (79). Microglia exhibit a hyperactivated phenotype characterized by elevated expression of MHC class II molecules and production of IL-6 and chemokines, which in turn aid T cell recruitment and expansion (79). Accordingly, NLRX1 function is predicted to attenuate the proinflammatory properties of microglia. On the other hand, NLRX1-deficiency has no intrinsic effect on the pool of encephalitogenic T cells (79). NLRX1 may also play a protective function in astrocytes; NLRX1^-/-^ astrocytes release excess glutamate in a Ca^2+^ dependent manner and contain reduced ATP levels compared to wild-type astrocytes, suggesting that NLRX1 promotes mitochondria ATP production (90). Furthermore, ROS levels in NLRX1 deficient astrocytes are increased compared to wild-type astrocytes, which may explain the reduced glutamate uptake (90). Recent evidence suggests that NLRX1 inhibits microglial activation in the early stages of EAE, which prevents activation of neurotoxic astrocytes (78).

NLRP12 has also been shown to regulate the progression and nature of CNS inflammation in EAE (87, 88, 153). NLRP12 mediates classical inflammasome driven inflammation in innate effector cells to certain microbes (164, 165), but also serves as a negative regulator of the NF-κB signaling pathway (80, 87, 88, 166, 167). In mice deficient of NLRP12, a more rapid and severe EAE develops (81). This exacerbated disease is characterized by increased mRNA levels encoding IL-1β and other proinflammatory molecules in the CNS, as well as activated microglia producing heightened levels of inducible NO synthase (iNOS), NO, TNFα, and IL-6 (81). A second study reported that EAE induction in NLRP12^-/-^ mice results in neuroinflammation that promotes ataxia and poor balance, rather than the ascending paralysis that normally develops in wild-type mice (87). Furthermore, NLRP12-deficiency has intrinsic effects on T cells. In the absence of NLRP12 expression, T cells exhibit increased proliferation, and secretion of IFNγ, IL-17 and IL-4, that is in part due to hyperactivation of NF-κB (87). Therefore, NLRP12 negatively regulates various aspects of innate cell activation, as well as CD4^+^ T cell expansion and effector function *via* blocking NF-κB signaling (88).

### Rheumatoid arthritis and inflammasome-mediated joint inflammation

RA is a chronic autoimmune disease characterized by the inflammation of the joints, leading to synovial tissue proliferation, cartilage erosion and joint destruction (168–170). Pathology is in part driven by Th1 and Th17 CD4^+^ T cells and B cells, as well as innate effectors such as monocytes, DC and neutrophils that traffick into the synovium (171–173). Joint-resident cells such fibroblast-like synoviocytes (FLS) also promote local inflammation (174). Normally, FLS play a key role in maintaining joint homeostasis *via* production of the extracellular matrix and matrix metalloproteinases (MMPs) (175).

**Figure 3:**
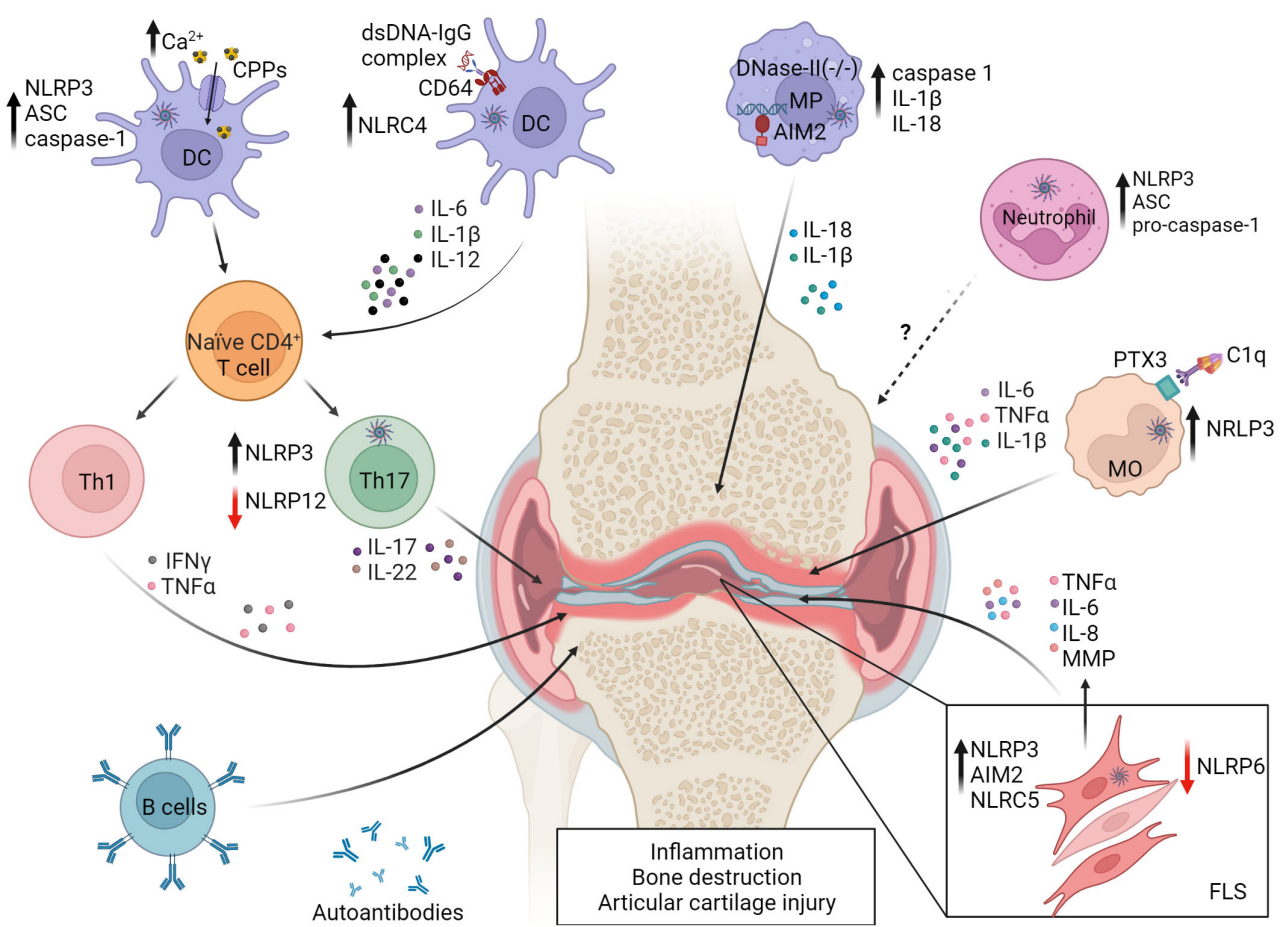
Events in dysregulated inflammasome activation in rheumatoid arthritis (RA). NLRP3 and NLRC4 activity are increased in monocytes (MO) and DC by Fc-receptor (FcR) binding of DNA-IgG immune complexes and complement component 1q (C1q) binding to pentraxin 3 (PTX3). Uptake of elevated levels of calciprotein particles (CPPs) in the joint by resident DC also leads to NLRP3 activation. Resulting pyroptosis and secretion of proinflammatory cytokines promote RA progression by favoring Th1 and Th17 differentiation and development of autoantibodies and RA factor producing plasma cells. NLRP3 activation is also increased in Th17 cells. Aberrant lysosomal processing of endocytosed dsDNA can lead to AIM2 activation in joint resident macrophages (MP). Neutrophils exhibit reduced expression of inflammasome molecules, which correlates with decreased disease severity. NLRP3, NLRC5 and AIM2 are associated with proinflammatory properties of fibroblast-like synoviocytes (FLS), while NLRP6 and NLRP12 serve protective roles, indicated by the red arrows. NLRP6 limits FLS cytokine production, and NLRP12 negatively regulates Th17 subset differentiation. Reduced expression of NLRP6 and NLRP12 leads to pathology. Matrix metalloproteinases (MMP). The figure was prepared using Biorender software licensed to the UNC Lineberger Comprehensive Cancer Center.

The autoimmune response of RA also involves high levels of serum complement and the production of autoantibodies that target the Fc region of IgG (i.e. rheumatoid factor), cartilage components, nuclear proteins and proteins post-translationally modified by citrullination (176, 177). Key proinflammatory cytokines driving RA include IL-1β and IL-18, as well as IL-6 and TNFα (178). In addition to having immunomodulatory effects, IL-1β mediates cartilage erosion and prevents chondrocyte matrix formation (179). Furthermore, the severity of RA correlates with elevated serum IL-18 (180, 181). Moreover, during the early-stages of RA, FLS proliferate and differentiate into distinct subsets of activated synovial fibroblasts that produce inflammatory cytokines, matrix-degrading enzymes and proangiogenic factors which lead to the release of inflammatory mediators, bone destruction and angiogenesis (182–184). FLS also promote T cell survival, Tfh and Th17 cell differentiation, and can function as antigen presenters to autoreactive T cells (185–193). The etiology of RA is ill-defined but genetic and a host of environmental factors are known to influence disease susceptibility and progression. Evidence also suggests that inflammasomes likely have an important role in RA pathogenesis (**Figure 3**).

In RA patients, NLRP3 and NLRP3-inflammasome-related proteins are upregulated in a cell-specific manner among innate effectors. For instance, expression of NLRP3, ASC, and caspase-1 as well as IL-1β secretion is generally increased in monocytes, macrophages, and DC from RA patients (99–102) (**Figure 3**). CD4^+^ T cells from RA patients also exhibit increased NLRP3 expression, which correlates with elevated serum IL-17A concentrations and disease activity (109) (**Figure 3**). Notably, differentiation of Th17 cells is inhibited by NLRP3 knockdown (109), suggesting that NLRP3 regulates the proinflammatory activity of both innate and adaptive effectors in RA. Interestingly, NLRP3 activation in monocytes is mediated *via* multiple mechanisms in RA patients. C1q binding to pentraxin 3, a key regulator of complement activity and which is increased on the surface of RA CD14^+^ monocytes, leads to NLRP3 activation, enhanced IL-1β and IL-6 secretion, and GSDMD-induced pyroptosis (178). In addition, due to elevated extracellular Ca^2+^ in the joint and concomitant heightened activity of calcium-sensitive receptors, macropinocytosis of calciprotein particles (CPPs) is elevated by local monocytes (194). After uptake, CPPs disrupt lysosome integrity resulting in enhanced NLRP3 activation and IL-1β secretion (194).

Whereas NLRP3 and related inflammasome proteins are typically elevated in various innate and adaptive immune effectors, neutrophils from RA patients exhibit reduced NLRP3, ASC and pro-caspase-1 expression (108). Here, *NLRP3* mRNA levels in neutrophils negatively correlate with disease severity (108). This suggests that NLRP3 may serve a protective role in the context of neutrophil function *via* an ill-defined mechanism (108).

Various inflammasome molecules, in addition to NLRP3, have been found to be involved with RA (**Figure 3**). NLRC4 activity is increased in DC residing in the synovial membrane of RA patients (105). These DC secrete elevated IL-1β, have increased expression of CD64, an IgG Fc receptor, and display an enhanced capacity to stimulate Th1 and Th17 subset differentiation (105). This capacity is due to a novel mechanism of upregulation of NLRC4 expression and activity. Here, dsDNA-IgG complexes bind to CD64, are internalized, and the combination of CD64 signaling and intracellular sensing of the dsDNA increases NLRC4 activity (105). AIM2 expression is increased in synovial tissue of RA patients, and knockdown of *AIM2* mRNA inhibits *in vitro* proliferation of FLS derived from RA patients (111). On the other hand, NLRP6 levels are reduced in FLS from patients with RA versus osteoarthritis (112). Furthermore, increased ectopic expression of NLRP6 in RA patient-derived FLS blocks the production of inflammatory cytokines such as IL-1β, IL-6, and TNFα, as well as MMP *via* inhibition of the NF-κB pathway. The latter indicates that NLRP6 serves a protective role in RA (112), and is consistent with NLRP6 having a negative regulatory function in colitis (195).

Animal studies further support the notion that the role for inflammasomes in RA is complex, and that cell type-dependent, inflammasome molecules can have distinct effects on immune cells and effector molecules depending on the RA model (103, 196) (**Figure 3**). Mice deficient of ASC are resistant to collagen induced arthritis (CIA), in part due to a reduced T cell stimulatory capacity of ASC^-/-^ DC (103). However, CIA develops in both NLRP3^–/–^ and Caspase1^–/–^ mice suggesting that ASC has caspase 1-independent effects in DC (103). On the other hand, NLRP3 and caspase-1 play a key role in the spontaneous polyarthritis that develops in mice in which the RA susceptibility gene *A20/Tnfaip3* is selectively ablated in myeloid cells (A20myel-KO mice) (104). Here, macrophages lacking A20 have increased constitutive and LPS-induced expression of NLRP3 and pro-IL-1β. The latter is indicative of the established role A20 has as an inhibitor of NF-κB activation (197), which is needed for NLRP3 and pro-IL-1β transcription following inflammasome priming. Furthermore, activation of NLRP3 in A20-deficient macrophages results in enhanced caspase-1 activation, IL-1β secretion, and pyroptosis. Notably, pathology in A20myel-KO mice is blocked by ablation of NLRP3, caspase-1 and the IL-1 receptor (IL-1R), demonstrating a direct role for classical NLRP3 inflammasome activation in this spontaneous autoimmune model of cartilage destruction (104). NLRP3 is also associated with the proinflammatory properties of FLS. NLRP3 expression is increased in FLS isolated from mice with adjuvant-induced arthritis (AA) (113), and knockdown of *Nlrp3* mRNA expression in FLS reduces disease severity in a monosodium urate-induced model of gout arthritis in rats (114).

AIM2 has also been shown to have a key role in joint inflammation. Mice deficient in expression of lysosomal endonuclease DNase II and type I IFN receptor (IFNαR) develop polyarthritis marked by production of autoantibodies, and macrophage secreted proinflammatory cytokines such as IL-1β, IL-6 and TNFα (106). Lack of lysosomal endonuclease DNase II results in aberrant processing of dsDNA in lysosomal compartments, and translocation of undigested DNA into the cytoplasm of macrophages (106, 107). AIM2-deficiency limits joint inflammation marked by reduced caspase-1 activity, IL-1β and IL-18 expression, and macrophage infiltration (106, 107). Notably, however, autoantibody production is unaffected by AIM2-ablation indicating a tissue-specific role for AIM2. Furthermore, AIM2-ablation has no effect on the transfer of arthritogenic serum from K/BxN mice (107). In this passive model, arthritis is induced by the deposition of immune complexes within the joint, leading to complement fixation and ensuing pathology (106, 107). Therefore, AIM2 regulates inflammation when cytosolic DNA is the key driving event. A contribution for NLRC5 in joint inflammation has been reported (115). NLRC5 expression is elevated in the synovium and FLS in rat AA (115), and knockdown of *Nlrc5* mRNA blocks FLS proliferation and production of TNFα and IL-6, due to suppressed NF-κB activation (115).

Similar to NLRP6, NLRP12 has been shown to negatively regulate joint inflammation (110). The severity of antigen-induced arthritis in NLRP12^-/-^ mice is increased, marked by elevated levels of joint infiltrating Th17 cells (110). Notably, *in vitro* Th17 cell differentiation is enhanced in NLRP12^-/-^ CD4^+^ T cells marked by elevated IL-6-induced activation of signal transducer and activator of transcription (STAT) 3 (110).

### Type 1 diabetes and inflammasome-mediated pancreatic islet inflammation

T1D is characterized by chronic inflammation of the pancreatic islets (insulitis) that results in the dysfunction and/or destruction of the insulin producing β cells (198–200). Despite life-long insulin therapy, T1D patients typically develop a variety of complications including retinopathy, neuropathy, and nephropathy related to hyperglycemia and inflammation. The autoimmune response involves islet infiltration of CD4^+^ and CD8^+^ T cells, B cells, macrophages, and DC. β cell-specific CD4^+^ and CD8^+^ T cells are generally believed to be the key drivers of pathology (198–200). Diabetogenic CD4^+^ and CD8^+^ T cells typically exhibit a type 1 effector phenotype, although Th17 cells are also implicated in the disease process (199). In addition to serving as APC, islet-infiltrating macrophages and DC, mediate β cell destruction through secretion of proinflammatory mediators and cytokines such as IL-1β, IFNγ and TNFα that have direct β cell-cytotoxic effects (199). The initiation and progression of T1D are influenced by genetic and poorly defined environmental factors (201–204). The latter include viral infections, and dysbiosis of gut microbiota, which are events that can be impacted by inflammasome activity (16, 201, 205).

**Figure 4:**
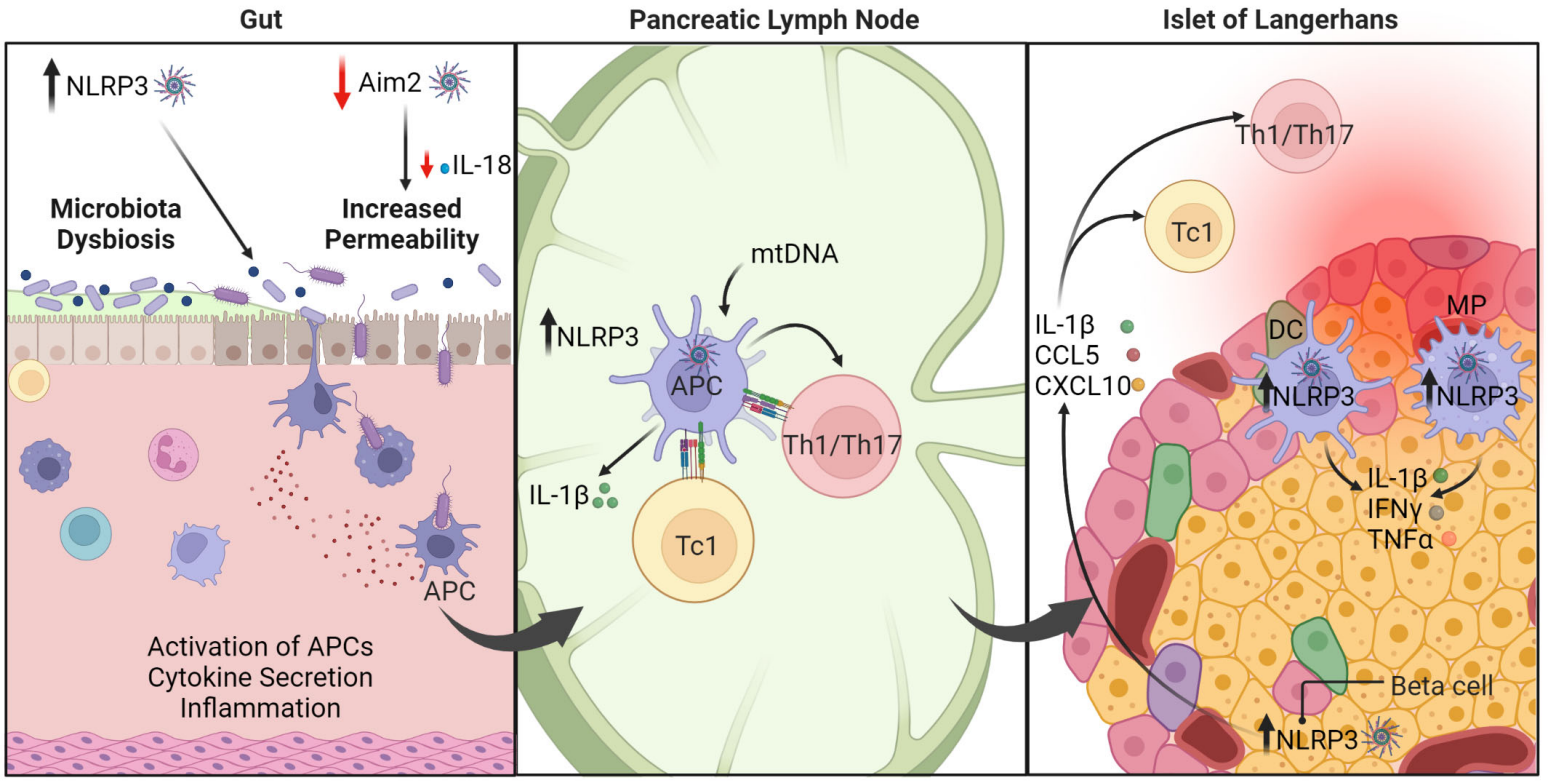
The roles of inflammasomes in type 1 diabetes (T1D). Under homeostasis, healthy intestinal epithelial cells maintain intestinal barrier function and regulate permeability to prevent passage of harmful elements such as microorganisms and toxins. AIM2 serves a protective function (indicated by the red arrow). Dysregulation of inflammasome function, such as AIM2 deficiency, leads to reduced production of IL-18, which is necessary for maintaining intestinal barrier function. Consequently, inflammasome dysregulation enhances intestinal permeability and triggers inflammation. On the other hand, NLRP3 is linked to dysbiosis within the gut microbiota, which can exacerbate T1D progression. In the pancreatic lymph node (PLN), upregulation of NLRP3 in APC promotes IL-1β production that ultimately drives differentiation of diabetogenic CD8^+^ Tc1, CD4^+^ Th1, and Th17 cells. In the pancreatic islets, NLRP3 hyperactivity in β cells induces release of cytokines and chemokines. These conditions combined with other immunomodulatory factors establish a positive feedback loop to further perpetuate pancreatic inflammation. Macrophage (MP), dendritic cell (DC), antigen-presenting cell (APC). The figure was prepared using Biorender software licensed to the UNC Lineberger Comprehensive Cancer Center.

Studies using murine models of T1D show that NLRP3 regulates the diabetogenic response (**Figure 4**). In non-obese diabetic (NOD) mice, which spontaneously develop β cell autoimmunity and overt diabetes, NLRP3 deficiency results in a reduced incidence of diabetes (123). This attenuated diabetes is due in part to NLRP3^-/-^ APC having a decreased capacity to promote Th1 cell differentiation; Th17 cell differentiation, however, is unaffected. Importantly, NLRP3^-/-^ β cells exhibit decreased production of IL-1β and chemokines such as CCL5, and CXCL10 (123). The latter limits migration into the islets by immune effectors including diabetogenic T cells (123) (**Figure 4**). Interestingly, limited IL-1β production leads to reduced activation of interferon regulatory factor 1 (IRF1) that is needed for β cell expression of CCL5 and CXCL10. Diminished IL-1β secretion by β cells is also expected to aid β cell viability and function, as well as enhance the maintenance and function of protective Foxp3^+^Treg in the islets. Notably, upregulation of NLRP3 and IL-1β is also detected in human β cells upon LPS and ATP stimulation *in vitro* (206). A regulatory function for NLRP3 in the disease process is also seen in a multiple low dose streptozotocin (MLD-STZ)-induced model of T1D. Here, progression of β cell autoimmunity is reduced in MLD-STZ treated C57BL/6 mice lacking NLRP3 expression (207). In this model NLRP3 is activated in macrophages residing in the draining pancreatic lymph nodes (PLN) by mitochondrial DNA (mtDNA) that is released following STZ treatment. NLRP3 activation results in increased caspase-1 activity, and IL-1β production, which drives expansion of pathogenic Th1 and Th17 cells and the induction of diabetes. The PLN are a key site for priming of diabetogenic CD4^+^ and CD8^+^ T cells. Interestingly, plasma levels of mtDNA are increased in T1D versus healthy subjects, which is expected to contribute to systemic inflammasome activation (208). Indeed, circulatory mtDNA induced by MLD-STZ in mice activates NLRP3 in endothelial cells *via* Ca^2+^ influx and mitochondrial ROS generation, which leads to endothelial dysfunction and vascular inflammation (208). Vascular inflammation is a key driver of complications that develop in T1D. Together these studies indicate that NLRP3 promotes pathological events driving β cell autoimmunity. Nevertheless, mechanisms by which NLRP3 mediate effects are likely to be complex and cell dependent. For instance, disease progression in NOD mice is unaffected by caspase-1 deficiency (209, 210), and only minimally affected by IL-1R ablation (211).

In contrast to NLRP3-deficient C57BL/6 (207), MLD-STZ enhances diabetes development in AIM2-deficient C57BL/6 mice (124). Interestingly, disease exacerbation in the AIM2^-/-^ mice is mediated by enhanced intestinal permeability, alterations in the gut microbiota, and increased bacterial translocation to the PLN where CD4^+^ Th1 and CD8^+^ Tc1 are readily expanded (**Figure 4**). Importantly, AIM2 deficiency results in decreased maturation of IL-18 which is needed to maintain intestinal barrier function (124). On the other hand, reduced NLRP3 expression in colonic NOD mouse tissue is associated with decreased microbiota dysbiosis, enhanced intestinal barrier function and diabetes prevention (125, 126). It is well established that dysbiosis within the gut microbiota significantly affects disease progression in NOD mice, and clinical findings suggest similar effects may also occur in T1D subjects (16, 205, 212–215). These studies provide evidence that inflammasomes may play a key role in regulating T1D progression in part *via* effects on gut microbiota and intestinal barrier function (16). Studies have reported that gut microbiota composition and/or intestinal barrier permeability are also influenced by other inflammasome molecules such as NLRP6 (216), NLRC4 (217), NLRX1 (218, 219), and NLRP12 (220, 221). Further investigation is necessary to elucidate the connection between inflammasomes, gut microbiota homeostasis, and autoimmunity.

### Systemic lupus erythematosus and the role of inflammasome activity in widespread inflammation

SLE is a chronic autoimmune disease with diverse clinical manifestations. Development of SLE is influenced by genetic, hormonal, and environmental factors that lead to dysregulation of mechanisms of innate and adaptive-mediated self-tolerance. The autoimmune response is characterized by the generation of anti-nuclear autoantibodies, tissue deposition of immune complexes, increased type I IFN production, and inflammation in multiple organs with the kidneys being the most commonly affected (222). CD4^+^ T cells such as Tfh cells are key drivers of the autoantibody response, and Th17 cells, found infiltrating the kidneys and skin contribute to tissue damage (223). Innate effectors such as monocytes, macrophages, DC and neutrophils also play roles in mediating the systemic inflammation and tissue damage in SLE (223).

**Figure 5:**
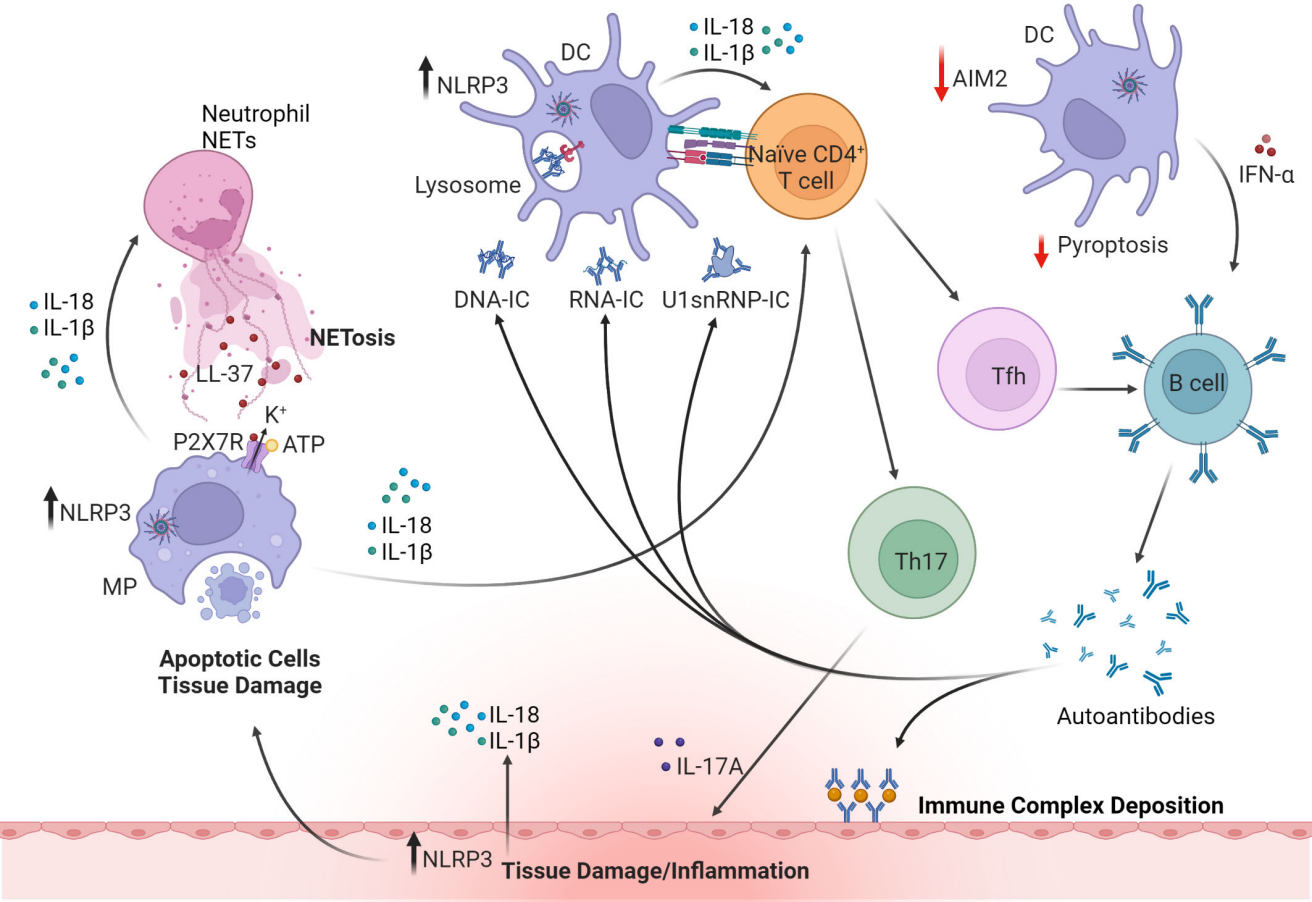
The roles of inflammasomes in systemic lupus erythematosus (SLE). Upregulation of NLRP3 inflammasome in macrophages (MP) and DC by DNA or RNA immune complexes (IC) or small nuclear ribonucleoprotein (snRNP) leads to release of proinflammatory cytokines such as IL-1β, IL-18 and IFNα. Dysregulation of inflammasomes in APC also promotes Th17 and Tfh cell differentiation. Tfh cells and IFNα facilitate B cell maturation and autoantibody production. However, production of IFNα is regulated by AIM2-mediated pyroptosis (indicated by red arrows). Deposition of IC, infiltrating Th17 cells, and production of autoantibodies and cytokines all contribute to tissue damage. IL-18 activates NETosis in neutrophils and in turn upregulates NLRP3 and IL-1β and IL-18 secretion in macrophages *via* cathelicidin antimicrobial peptide (LL37)-driven K^+^ efflux mediated by the P2X7 receptor (P2X7R). These cytokines further induce pyroptosis and release of cellular and nuclear contents, leading to the production of anti-nuclear autoantibodies and further amplifying systemic inflammation. Inflammasome activation in cells of target tissues, such as kidney resident podocytes also contributes to disease pathology by producing IL-1β. The figure was prepared using Biorender software licensed to the UNC Lineberger Comprehensive Cancer Center.

The etiology of SLE is not fully understood but evidence from humans and animal models indicate that inflammasomes contribute to disease progression (**Figure 5**). Inflammasome components are typically upregulated in kidney biopsies from SLE patients, and NLRP3, IL-1β and IL-18 are increased in SLE patient macrophages, peripheral blood mononuclear cells (PBMC), and serum (133, 134). A critical meditator of pathology in SLE are anti-nuclear autoantibodies (ANA) that target endogenous dsDNA and ribonucleoproteins (RNP) (224). Immune complexes (IC) of dsDNA upregulate NLRP3 and caspase-1 activity leading to increased IL-1β production by monocytes and macrophages of SLE patients (225). Here, the IC activates TLR9, a DNA sensor, which subsequently upregulates NF-κB and primes inflammasome assembly *via* increasing NLRP3 and pro-IL-1β (225). Upon IC binding, TLR9 also promotes mitochondrial ROS production and K^+^ efflux and subsequent NLRP3 activation. Notably, SLE monocytes stimulated with dsDNA-antibody complexes readily promote differentiation of Th17 cells, which is also seen *in vivo* in lupus-prone NZBW/F1 mice injected with anti-dsDNA autoantibodies from SLE patients (224). Similarly, autoantibody complexes of U1-small nuclear RNP (U1-snRNP) activate the NLRP3 inflammasome involving cytoplasmic RNA sensors TLR7 and TLR8 signaling in human monocytes (226). Antibody complexes of endogenous snRNP also induce production of macrophage migration inhibitory factor (MIF) in human monocytes, which enhances NLRP3 activation and IL-1β production (227). Interestingly, the context of nucleic acid uptake appears to determine the identity of the inflammasome molecule being engaged. For instance, unbound dsDNA, normally found at high levels in SLE patient serum, is taken up by monocytes *via* macropinocytosis, which activates AIM2 as well as NLRP3 (135). Uptake of free nucleic acid, however, requires antibody to be internalized by macropinocytosis but not Fc receptor (FcR) (135). On the other hand, internalization of dsDNA/snRNP autoantibody complexes *via* FcR may favor activation of NLRP3, and possibly NLRC4 as seen in RA (105). In each of the aforementioned scenarios, IL-1β and IL-18 are secreted to maintain/amplify inflammation. Furthermore, induced pyroptotic death and release of cellular and nuclear contents lead to the production of ANA to further fuel the autoimmune response (228, 229).

Aberrant clearance of neutrophil extracellular traps (NETs) is also linked with the pathogenesis of SLE and inflammasome activation (**Figure 5**). NETs are a network of chromatin fibers containing anti-microbial peptides such as LL37 and enzymes that participate in host defense (230). NETs are primarily released by activated neutrophils that undergo NETosis, a programmed cell-death mechanism (231). Notably, NETs activate NLRP3 inflammasome and IL-1β and IL-18 secretion in macrophages from SLE patients *via* LL37-driven K^+^ efflux mediated by the P2X7R (136). Furthermore, IL-18 activates NETs and promotes NETosis suggesting that a feed-forward loop exists that helps to maintain inflammation (136).

Monocytes from SLE patients versus healthy controls exhibit enhanced NLRP3 activation and IL-1β secretion (138, 139). This hyperactivity is attributed to chronic IFNα stimulation of monocytes. Elevated type I IFN-induced gene expression “signatures” correlate with the presence of autoantibodies, nephritis, and disease activity (232). Prolonged IFNα exposure *in vivo* induces NLRP3 hyperactivity by an IRF1 signaling pathway (138). However, consistent with other studies (233), short-term IFNα exposure of monocytes blocks NLRP3 activation (138). The latter, importantly, indicates that chronic type I IFN stimulation can have distinct effects on inflammasome activation.

The study of different murine lupus models provides further evidence that inflammasomes regulate SLE pathogenesis. Mice deficient in caspase-1 expression versus wild-type mice exhibit reduced autoantibody production, a limited IFN signature, as well as diminished NETosis and kidney pathology induced by pristane administration (136). In addition, blocking the P2X7R significantly impacts the development of spontaneous lupus in MRL/lpr mice. Here, limiting NLRP3 activation reduces the production of anti-dsDNA autoantibodies and IL-1β, and decreases Th17 cell expansion and the severity of nephritis (234). Furthermore, various drugs that inhibit NLRP3 inflammasome activation attenuate disease severity in different lupus mouse models (137, 235–237). On the other hand, nephritis induced by pristane treatment is exacerbated in mice in which myeloid cells selectively express a transgene encoding a hyperactive *Nlrp3^R258W^* mutant protein (238).

In addition to immune effector cell types, inflammasome activation in target tissues also contributes to disease pathology (**Figure 5**). Endothelial cells, basement membrane, and podocytes form a glomerular filtration barrier, which is essential for maintaining kidney function (239). In NZM2328 mice, which spontaneously develop lupus nephritis, severe proteinuria correlates with increased activation of NLRP3 and caspase-1 as well as IL-1β secretion by glomerular podocytes (141, 142). NZM2328 mice treated with MCC950, an NLRP3 inhibitor, exhibit reduced NLRP3 activation by podocytes, and attenuated renal tissue damage and proteinuria (141, 142).

Depending on the lupus model, inflammasome molecules have also been shown to play a protective role. In C57BL/6^lpr/lpr^ mice, which develop mild lupus, deficiency of NLRP3 or ASC exacerbates pathology marked by an increase in activated macrophages and DC and production of proinflammatory cytokines, and T and B cell proliferation but no effect is seen on autoantibody production (240). This enhanced pathology is marked by reduced SMAD2/3 phosphorylation during TGF-β receptor signaling, and consistent with the role of TGF-β1 as a key regulator of immune homeostasis (240). In this scenario, it is likely that NLRP3 or ASC serve functions independent of classical inflammasome activation (see below), consistent with the observation that IL-1R- or IL-18-deficiency in C57BL/6^lpr/lpr^ mice does not exacerbate pathology.

Studies have indicated that AIM2 may also serve a protective role in lupus by negatively regulating type I IFN production. In B6.Nba2 mice, which spontaneously develop lupus nephritis, p202, another IFN-inducible p200 family member is up-regulated (241, 242). Notably, p202 blocks AIM2 inflammasome assembly, and pyroptosis-mediated cell death. Consequently, p202 or other dsDNA sensors such as cyclic GMP-AMP synthase (cGAS), bind cytosolic DNA to promote prolonged type I IFN production that would be normally terminated by AIM2-induced pyroptosis (243). Regulation of pyroptosis has also been found to impact other aspects of the autoimmune response driving lupus nephritis. Pristane-induced lupus nephritis is exacerbated in mice lacking T cell expression of the P2X7R (140). Here, the P2X7R normally mediates GSDMD-driven pyroptosis of Tfh cells, which then limits differentiation of autoantibody secreting plasma cells in the germinal centers. Together these findings demonstrate the complexity of the roles inflammasomes have in both promoting and suppressing the autoimmune response of SLE.

## Alternative roles of inflammasome molecule-mediated regulation

Classical inflammasome activation and induction of a proinflammatory response contributes to autoimmunity in a variety of ways as described above. It is becoming apparent, however, that inflammasome molecules also serve regulatory functions independent of typical inflammation-driving events (**Table 2**). Caspase-1 for instance, in addition to being involved in the maturation of IL-1β and IL-18, has been shown to modulate protein secretion, cell death, and lysosomal function in many cell types such as neurons, hepatocytes, epithelial cells, and cardiomyocytes (244–251). These alternative roles for inflammasome molecules have been linked to regulation of immune effector cells such as T and B cells, as well as non-immune tissue-resident cell types. Accordingly, some of these events have been reported to be directly involved in the progression of autoimmunity, and/or can be expected to contribute to an autoimmune response.

**Table 2:** Alternative functions of inflammasome molecules in autoimmunity.

Inflammasome involved	Alternative mechanism	Associated diseases
**ASC**	Affects Th17, IL-1β maturation	MS
**NLRP3**	↑Th2 differentiation	MS
	↑TGF-β signaling	SLE
**AIM2**	↓Microglia inflammation	MS
	↑AIM2 in astrocytes in EAE model Maintains Foxp3^+^Treg function Regulates Th1/Th17 differentiation	
	↑Tfh differentiation	SLE
	↑AIM2 in GC B cell, memory B cells, and plasma cells from SLE patients	

Multiple sclerosis (MS); experimental autoimmune encephalomyelitis (EAE); systemic lupus erythematosus (SLE); germinal center (GC).

“↑” indicates increased activity of a given molecule. “↓” indicates reduced activity of a given molecule.

### ASC: A regulatory function in CD4^+^ T cells

ASC has a T cell intrinsic effect regulating the production of IL-1β needed to maintain CNS-resident Th17 cells in EAE. Recent findings indicate that ASC also regulates properties of murine CD4^+^ T cells independent of classical inflammasome activation and IL-1β maturation (252). ASC is constitutively expressed in naïve CD4^+^ T cells, and after anti-CD3/CD28 antibody stimulated TCR signaling, ASC is upregulated but no IL-1β or IL-18 secretion is detected (252). Naïve CD4^+^ T cells lacking ASC expression normally differentiate *in vitro* into Th1, Th2, Th17, Th9, and Foxp3^+^Treg subsets under polarizing conditions (252). Notably, recombination activation gene (Rag)-deficient mice develop more severe colitis after transfer of ASC^-/-^ CD4^+^ T cells versus wildtype, NLRP3^-/-^, or Caspase1^-/-^ CD4^+^ T cells (252). This increased pathogenic function of ASC^-/-^ CD4^+^ T cells is marked by enhanced TCR signaling *in vitro*, elevated lymphopenic proliferation *in vivo*, and an increased metabolic state marked by higher glycolytic flux and increased glucose transporter 1 (Glut-1) surface expression (252). These findings suggest a negative regulatory function for ASC in CD4^+^ T cell TCR signaling, proliferation, and metabolism. The mechanism(s) by which ASC regulates these events still needs to be defined. Nevertheless, one could envision a scenario in which dysregulation of alternative ASC function enhances the pathogenic potential of autoreactive CD4^+^ (and possibly CD8^+^) T cells to aid autoimmune disease progression.

### NLRP3 and Th2 cell differentiation

NLRP3 has also been found to have T cell-intrinsic effects independent of classical inflammasome activation. Specifically, NLRP3 positively regulates Th2 subset differentiation (253). Upon TCR stimulation by anti-CD3/CD28 antibody, expression of NLRP3 is increased in both Th1 and Th2 cells, due in part to IL-2 induced STAT5 activity (253). However, NLRP3-deficiency reduces Th2 but not Th1 cell differentiation (253). Importantly, ASC or caspase-1 deficiency has no effect on NLRP3-mediated Th2 lineage differentiation ruling out a role for classical NLRP3 inflammasome activity (253). Findings indicate that NLRP3 functions as a transcription factor regulating *Il4* transcription (253). Here, NLRP3 forms a complex with the transcription factor IRF4, that enhances the binding of the IRF4 to the *Il4* promoter; however, NLRP3 alone is insufficient to mediate *Il4* transcription (253). Notably, induction of asthma, which is Th2 cell-dependent, is reduced in NLRP3-deficient mice (253). Furthermore, NLRP3^-/-^ mice also more readily reject implanted B16F10 tumor cells due to an elevated Th1 cell response (253). In wildtype recipients, increased differentiation of Th2 cells permits the progression of B16F10 tumors (253). In the case of autoimmunity, aberrant Th2 cell differentiation has been associated with skewed development of Th1 and Th17 cells, which drive the pathology in MS, RA, T1D and SLE (254). Accordingly, aberrant expression and/or function of NLRP3 that is independent of inflammasome activity, may favor the development of pathogenic autoreactive Th1 and Th17 effectors. For instance, reduced IL-2 signaling and STAT5 activation, which is associated with T1D (255), would be expected to limit *Nlrp3* transcription and Th2 cell differentiation.

### Roles of AIM2 independent of inflammasome activation

Studies demonstrate that AIM2 displays a number of alternative functions independent of inflammasome activation in various cell types, that affect the progression of autoimmunity. Recently, AIM2 was shown to have a T cell-intrinsic role in regulating peripheral Foxp3^+^Treg (256). AIM2 is highly expressed in murine and human Foxp3^+^Treg, and AIM2 expression is upregulated by TGF-β1 stimulation (256). TGF-β1 is required for peripheral differentiation of CD4^+^ T cells into Foxp3^+^Treg (257). In AIM2-deficient C57BL/6 mice, MOG_35-55_-induced EAE is exacerbated characterized by increased Th1 and Th17 cell infiltration, and a reduction in the frequency of Foxp3^+^Treg in the CNS (256). A diminished local pool of Foxp3^+^Treg favors the expansion and effector function of encephalitogenic Teff (256, 257). Foxp3^+^Treg are unaffected by ASC-deficiency, indicating that the role for AIM2 is inflammasome-independent (256). Notably, AIM2 in Foxp3^+^Treg attenuates AKT activation, and downstream mTOR and MYC signaling that leads to glycolysis (256). Normal Foxp3^+^Treg differentiation and lineage maintenance is achieved under metabolic conditions favoring oxidative phosphorylation of lipids (256). On the other hand, glycolysis negatively impacts Foxp3^+^Treg stability and function (256). AIM2 serves to maintain Foxp3^+^Treg under proinflammatory conditions by forming a complex consisting of the adaptor protein receptor for activated C kinase 1 (RACK1), and the protein phosphatase 2 (PP2A) phosphatase that blocks AKT phosphorylation (256).

AIM2 has also been reported to regulate Tfh independent of inflammasome activation (258). Tfh from blood and skin lesions of SLE patients express elevated levels of AIM2. In mice in which AIM2 is conditionally ablated in T cells, the severity of pristane-induced lupus nephritis is reduced relative to control animals. The latter corresponds with a decreased Tfh pool. Notably, AIM2 regulates Tfh differentiation through an interaction with transcription factor c-MAF, that in turn is needed to promote *Il21* gene transcription (258). Interestingly, *Aim2* mRNA expression is upregulated by IL-21 stimulation suggesting that AIM2 participates in a feed-forward loop promoting Tfh differentiation and function.

In addition to T cells, AIM2 has been shown to have a B cell-intrinsic effect independent of inflammasome activation. SLE patients exhibit elevated AIM2 expression in germinal center (GC) B cells, memory B cells and antibody secreting plasma cells prepared from the tonsils, blood and/or skin lesions (259). Furthermore, pristane-induced lupus nephritis is attenuated in mice in which AIM2 is conditionally ablated in B cells. Limited disease is reflected by diminished numbers of GC B cells, and plasma cells. Findings suggest that AIM2 is an upstream regulator of the Blimp1-BCL6 transcriptional axis, which drives GC B cell and plasma cell differentiation (259).

AIM2 also serves a protective role in EAE by limiting the inflammatory properties of brain-resident microglia (151). Whereas ASC-deficiency in mice attenuates EAE as discussed above, AIM2-deficiency exacerbates EAE severity. Furthermore, selective ablation of AIM2 in microglia is sufficient to enhance the encephalitogenic response. In microglia, AIM2 negatively regulates a proinflammatory phenotype by suppressing the activity of DNA-dependent protein kinase (DNA-PK) and downstream activation of AKT3. Inhibition of AKT3 reduces phosphorylation of the key transcriptional factor IRF3, which blocks the production of chemokines, type I IFN, and the expression of antigen presentation molecules by microglia (151). AIM2 similarly inhibits DNA-PK and AKT activation in colon epithelial cells to protect mice from colitis and colon cancer (260). Interestingly, a recent study provides evidence that AIM2 has an alternative role in an EAE model independent of robust classical inflammasome activation (152). Through the use of a novel reporter mouse to track inflammasome activation *in situ*, AIM2 activation is seen to be prevalent in astrocytes but not CNS infiltrating monocytes and macrophages. Despite elevated AIM2 expression, no marked *Il1b* expression and cell death are detected in astrocytes (152). The role of AIM2 in this scenario needs to be further defined.

## Targeting inflammasome molecules to prevent/treat autoimmunity

Inflammasome molecules offer an appealing target for immunotherapy and the treatment of autoimmunity. Several inhibitors targeting inflammasome-related molecules have been identified, developed, and tested in preclinical studies or clinical trials (**Table 3**). MCC950, a small-molecule inhibitor, specifically binds to the Walker B motif of the NACHT domain of NLRP3 to block function (287). Therapeutic efficacy and safety of MCC950 and analogs (Inzomelid and Somalix) have been assessed in several preclinical studies with promising results (288–294) (TrialTrovelID-368867; TrialTrovelID-360928). Nevertheless, a phase II clinical trial for RA showed that MCC950 has safety concerns related to elevated serum liver enzyme levels. Other NLRP3 inhibitors are currently being evaluated in animal studies of EAE (264, 266, 272, 279).

**Table 3:** Therapeutic strategies targeting inflammasomes for autoimmunity.

Targeted inflammasome-associated molecule	Therapeutic	Disease	Ref
**Upstream signal of NLRP3**	Disulfiram	MS	(261)
**NLRP3**	MCC950	MS	(262)
		RA	(99)
		SLE	(141)
	1,2,4-trimethoxybenzene	MS	(263)
	OLT1177	MS	(264)
		RA	(265)
	RRx-001	MS	(266)
	JC171	MS	(267)
	Tranilast	RA	(268)
	A20	RA	(104)
	Curcumin	SLE	(236)
	Melatonin	SLE	(137)
	Piperine	SLE	(269)
	Citral	SLE	(270)
**AIM2**	Myricitrin	RA	(271)
**ASC**	IC100	MS	(272)
	Lonidamine	MS	(273)
	Spirodalesol analog 8A	MS	(274)
**Caspase-1**	VX-765	MS	(275)
	VX-740	RA	(276)
**GSDMD**	Necrosulfonamide	MS	(277)
	DMF	MS	(278)
**NF-κB**	BAY11-7082	MS	(279)
		SLE	(280)
	Methotrexate	RA	(281) NCT04464642
	Icariin	SLE	(282)
**Interleukin-1**	Anakinra	RA	(283)
	Canakinumab	RA	(284)
		T1D	(285)
	Gevokizumab	T1D	(286)

Multiple sclerosis (MS); rheumatoid arthritis (RA); type 1 diabetes (T1D); systemic lupus erythematosus (SLE); Gasdermin D (GSDMD).

Caspase-1 is another key target for therapeutic intervention of autoimmunity. VX-765 (belnacasan), a caspase-1 inhibitor, blocks GSDMD-mediated pyroptosis, reduces inflammasome-associated proteins in the CNS, and attenuates EAE in mice (275). However, testing of the related caspase-1 inhibitor VX-740 was discontinued in a RA clinical trial due to the liver toxicity observed in animal models (295). Inhibiting GSDMD by necrosulfonamide reduces neuroinflammation and necroptosis in collagenase VII-induced mouse intracerebral hemorrhage model (277). In addition, dimethyl fumarate, an immunosuppressive drug used for the treatment of recurrent remission MS and plaque psoriasis promotes succination of GSDMD, which in turn disrupts the interaction with caspase-1 and blocks pyropotosis (278). Disulfiram, a drug used for alcohol addiction treatment, blocks pore formation by targeting Cys191/Cys192 in GSDMD (261).

IL-1β, which is associated with the pathogenesis of several autoimmune diseases, has been therapeutically targeted. Two FDA-approved biologics that block IL-1 activity have been clinically tested. Anakinra is a recombinant human IL-1R antagonist mainly applied for the treatment of RA. Due to a short half-life and low response rate compared to other treatments available, the usage of anakinra is limited, and efficacy is selective. For example, anakinra shows no efficacy for the treatment of T1D and Sjogren’s disease. Canakinumab is an anti-IL-1β neutralizing monoclonal antibody and has shown efficacy in RA and systemic juvenile idiopathic arthritis but no benefit for recent onset T1D patients (285, 296). IL-18 blockers have also been established but have not been applied for the treatment of autoimmunity.

## Summary/conclusions

The evidence at hand establishes roles for classical inflammasome activated inflammation and alternative pathways regulated by inflammasome molecules in autoimmunity. Inflammasome molecules have been implicated in human MS, RA, T1D and SLE, and shown in corresponding disease models to override and/or maintain self-tolerance (**Table 1**). Intrinsic and extrinsic effects on both APC and other innate effectors as well as T and B cells enables inflammasome molecules to establish the nature and specificity of an autoimmune response. Similarly, inflammasome molecules have intrinsic and extrinsic effects that alter the cellular integrity of tissues, independent of immune effectors. In a given tissue, inflammasome activity can impact inflammation by initiating and/or further driving a local autoimmune response, which in turn may be influenced by induction of pyroptosis versus PANoptosis cell death pathways. Alternatively, dysregulated inflammasome function can have more broad effects. This is seen with aberrant inflammasome activity reducing intestinal barrier function, which results in shifts within the microbiota composition that can impact the production of systemically released metabolites and favor proinflammatory versus immunoregulatory events (214).

The key events that drive inflammasome molecule activity in autoimmunity are poorly understood. What is apparent, however, is that multiple pathways and mechanisms exist to induce activation, in part reflecting the specificity of different inflammasome molecules. Poorly understood environmental factors known to influence MS, T1D, RA and SLE are likely involved in inducing inflammasome molecule activity. Release of PAMPs due to microbial infections or DAMPs due to cytotoxic effects of drugs, toxins, or UV irradiation for example, are obvious candidates to engage classical inflammasome-mediated inflammation. Polymorphisms in various inflammasome genes may also contribute to the polygenic influence on the development of MS, T1D, RA and SLE. Genetic analyses show that single nucleotide polymorphisms (SNPs) in genes encoding sensor molecules (i.e. *NLRP1*, *NLRP3*, *AIM2*) and inflammasome-related proteins (i.e. *PYCARD*, *CASP1*) are linked with susceptibility to and/or response to therapy for MS, T1D, RA and SLE (75, 78, 98, 119, 120, 153, 297–303). However, whether the disease-linked SNPs override the normally tight regulation of gene expression and/or function of inflammasome molecules needs to be ascertained. Inflammasome activity is also the consequence of collateral damage induced by autoimmunity. Autoimmune-mediated cytotoxicity leads to the release of DAMPs and a proinflammatory *milieu* induces local cellular stress affecting metabolism and mitochondrial function for instance, that drive inflammasome molecule activity.

The relative contribution(s) inflammasome molecule activity has in autoimmunity is poorly understood. Questions of whether inflammasome molecules mediate initiating events and/or modulate the progression and severity of autoimmunity need to be addressed. Environmental insults have typically been proposed to initiate autoimmunity where inflammasome activation is likely to occur (**Table 1**). Alternatively, sterile inflammation driven by metabolically stressed cells may stimulate dysregulated inflammasome activity and initiate autoimmunity. Pancreatic β cells are susceptible to metabolic stress due high levels of insulin expression and secretion (304, 305) that may lead to NLRP3 activation, for example. Reports showing that inflammasome expression and activity are upregulated in MS, T1D, RA and SLE patients suggest a role in at least supporting disease progression. Feed-forward loops in which inflammasome molecule activity are self-sustaining as well as promoting autoimmune reactivity and *vice versa* have been described. The use of murine models of spontaneous autoimmunity coupled with cell-specific and inducible expression systems will be helpful in further defining the contribution in the disease process for a given inflammasome molecule.

Of keen interest moving forward is defining regulation of inflammasome molecule-mediated events that are independent of classical activation of inflammation (**Table 2**). A hint to the complexity that is involved is exemplified by AIM2. As discussed above AIM2 regulates peripheral Foxp3^+^Treg differentiation by blocking AKT signaling through a AIM2-RACK1-PP2A complex (256). On the other hand, AIM2 suppresses colon carcinoma by binding to and inhibiting DNA-PK and downstream AKT signaling events needed for colon epithelial cell transformation (260). Therefore, depending on the cell-type, AIM2 inhibits PI3K-AKT signaling but *via* distinct complexes and mechanisms. Furthermore, AIM2 is reported to interact with the c-MAF transcription factor to positively promote Tfh differentiation (258). The nature of the signaling events that stimulate alternative inflammasome molecule activity, and the outcome of that activity in immune and non-immune cell types are important issues that require continued investigation.

To date, the therapeutic benefit of inhibiting inflammasome activation has mostly been demonstrated in animal disease models with limited success in the clinic (**Table 3**). The general lack of efficacy may reflect the timing and relative contribution of an inflammasome molecule in a given autoimmune disease. For instance, inflammasome activation may play a prominent role early in a disease process. Therefore, targeting inflammasome activity once an autoimmune response is well established, which is typical in the clinic, may have only a minimal effect. There is the important concern that inhibiting a given inflammasome molecule, particularly long-term, may compromise immunity against pathogens. Therefore, both efficacy and safety may be enhanced by combining an inflammasome-based approach with other types of immunotherapies. For example, limiting ongoing inflammation by blocking inflammasome activity may enhance the efficacy of antigen-based immunotherapy and induction of protective Treg.

The etiology of MS, T1D, RA and SLE is highly complex, and ill-defined. Establishing the roles of inflammasome activity in autoimmunity will aid our understanding of the mechanisms that drive these disease processes, as well as provide the impetus for the development of novel strategies of immunotherapy for disease prevention and treatment.

## Author contributions

QK, AG, VM, XJ and RT contributed to the preparation of the review article. All authors contributed to the article and approved the submitted version.
